# Validation of MR-Based Attenuation Correction of a Newly Released Whole-Body Simultaneous PET/MR System

**DOI:** 10.1155/2019/8213215

**Published:** 2019-11-28

**Authors:** Guobing Liu, Tuoyu Cao, Lingzhi Hu, Jiaxu Zheng, Lifang Pang, Pengcheng Hu, Yushen Gu, Hongcheng Shi

**Affiliations:** ^1^Department of Nuclear Medicine, Zhongshan Hospital, Fudan University, Shanghai 200032, China; ^2^Shanghai Institute of Medical Imaging, Shanghai 200032, China; ^3^Institute of Nuclear Medicine, Fudan University, Shanghai 200032, China; ^4^Shanghai United Imaging Healthcare Co., Ltd., Shanghai 201807, China

## Abstract

The aim of this study was to validate quantitative performance of a newly released simultaneous positron emission tomography (PET)/magnetic resonance imaging (MRI) scanner, by using MR-based attenuation correction (MRAC), both in phantom study and in patient study. PET/MRI image uniformities of a phantom under different hardware configurations were tested and compared. Thirty patients were examined with 2-deoxy-2-[^18^F]fluoro-D-glucose (^18^F-FDG) PET/computed tomography (CT) and subsequent PET/MRI. PET images from PET/MRI were corrected with MRAC (PET_MR_), CT-based attenuation maps (*μ*-maps, PET_CT_), and segmented CT *μ*-maps (PET_CTSeg_) derived from PET/CT. Standardized uptake values (SUVs) were compared among the 3 sets of PET in main organs (bone, liver and lung) and in 52 FDG-avid lesions, including soft-tissue lesions and bone lesions. The result showed that PET imaging uniformities of PET/MRI under different configurations were good (<8.8%). The SUV differences among the 3 sets of PET varied with organs and lesion types. In detail, the mean relative differences of SUV between PET_MR_ and PET_CT_ were as follows: −18.8%, bone (SUV_mean_); −8.0%, liver (SUV_mean_); −12.2%, lung (SUV_mean_); −18.1%, bone lesions (SUV_mean_); −13.3%, bone lesions (SUV_max_); −8.2%, soft-tissue lesions (SUV_mean_); and −7.3%, soft-tissue lesions (SUV_max_). The mean relative differences between PET_MR_ and PET_CTSeg_ were as follows: −19.0%, bone (SUV_mean_); −3.5%, liver (SUV_mean_); −3.3%, lung (SUV_mean_); −19.3%, bone lesions (SUV_mean_); −17.5%, bone lesions (SUV_max_); −5.5%, soft-tissue lesions (SUV_mean_); and −4.4%, soft-tissue lesions (SUV_max_). The differences of SUV between PET_MR_ and PET_CT_ were larger than those between PET_MR_ and PET_CTSeg_, in both soft tissue and soft-tissue lesions (*P* < 0.001), but not in bone or bone lesions. In conclusion, MRAC in the newly released PET/MR system is accurate in most tissues, with SUV deviations being generally less than 10%, compared to PET/CT. In bone, however, underestimations can be substantial, which may be partially attributed to segmentation of the MR-based *μ*-maps.

## 1. Introduction

The integrated whole-body positron emission tomography (PET)/magnetic resonance imaging (MRI) is emerging as a potential tool in clinical practice and in medical research. To date, there have been three commercial PET/MRI systems introduced; the first system was launched by Siemens (mMR PET/MR) in 2010 featuring an integrated design for simultaneous acquisition, the second system was released by Philips (Ingenuity TF PET/MR) in 2011 using a rotating table for sequential acquisitions, and most recently, a second simultaneous PET/MR system was introduced by GE (SIGNA PET/MR) in 2016 [1–3].

In 2018, the United Imaging Healthcare Corporation (Shanghai, China) released a fourth system (uPMR790) to be sold commercially. This system comprises a 3T superconducting magnet, a gradient system with a set of second-order active shimming coil (50 mT/s, 200 T/m/s), and a 48-channel radio frequency (RF) receiving system. The PET detector is installed between the gradient coil and body coil, which comprises 20 modules with a transverse field of view (FOV) of 60 cm and an axial FOV of 32 cm. Each module contains 5 × 14 blocks, and each block has 4 SiPM detector channels coupled with a 7 × 8 array of 15.5 × 2.76 × 2.76 mm^3^ LYSO crystals through the proprietary design of internal light guide. The entire system comprises 112 rings, and each ring contains 700 crystal channels, making 78400 crystal channels in total. In short, the uPMR790 features 2.8 mm PET spatial resolution, the highest resolution to date, and the longest axial FOV (32 cm) of all PET/MR systems. The PET component of PET/MR systems remains challenging due to hardware integrations, and aspects of PET image reconstruction, especially attenuation correction, still remain as an issue. Therefore, performance validation of this system is needed before putting it into clinical use.

Interpretation of the PET images always requires quantification of the tracer distribution, which heavily relies on attenuation correction (AC) during image reconstruction. However, the MR-based AC (MRAC) is not straightforward and quite different from computed tomography- (CT-) based AC (CTAC), as signal intensity of MRI does not reflect the electron density of the tissue, rendering a direct transformation of signal intensity to linear ACs (LACs) impossible [4–6]. To solve this problem, several approaches have been developed for MRAC, of which the segmentation-based methods have been commonly used and implemented into vendor-provided software [5, 7]. These methods separate the human body into several different tissue types usually using a T1-weighted 3-dimensional gradient-echo sequence with 2 echoes for fat and water separation. Afterward, predefined LACs are used for the different tissue types, allowing for computation of an attenuation map (*μ*-map) [5, 6, 8].

Several studies compared the segmentation-based MRAC against the well-established CTAC [7, 9–16]. However, conflicting results regarding underestimation or overestimation in SUVs of certain tissues remain. Furthermore, all of these comparisons were performed directly between the segmented MRAC and the continuous CTAC. Therefore, the results and comparisons inevitably suffered from the impact of segmentation to the MRAC data. In addition, nonpatient objects such as MR coils within the PET FOV could also affect the accuracy of attenuation correction [9, 15, 17]. Therefore, it is necessary to exclude the impact from these factors before validating the accuracy of the MRAC method.

This study aims at validating the performance of a simultaneous whole-body PET/MR system through two steps. In the first step, we investigated the impact of the hardware components of PET/MRI scanner (i.e., the track, patient bed, and MR coils) on the MRAC method based on the phantom study. In the second step, a volume-of-interest (VOI) based approach was conducted to validate the quantification accuracy of the MRAC-based PET in different tissues and lesions through patient-based study.

## 2. Materials and Methods

### 2.1. Phantom Study

**Figure 1:**
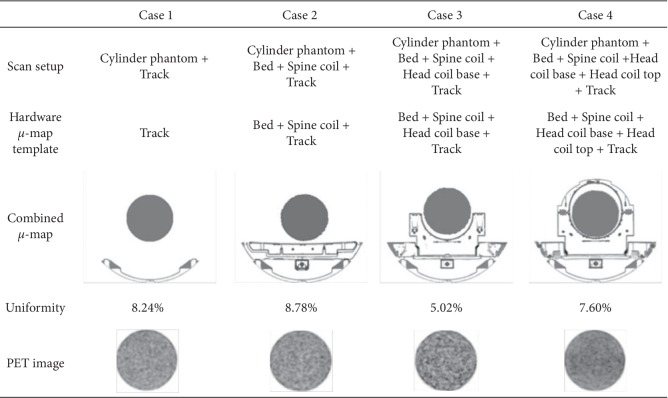
Image uniformity under different MR hardware configurations.

A uniform cylinder phantom (20 cm in diameter) filled with 1 mCi ^68^Ge was imaged at the center of a whole-body simultaneous PET/MR system (uPMR 790, United Imaging Healthcare, Shanghai, China). Data were acquired under four different configurations (Figure 1): (1) with the track of patient bed; (2) with the track, patient bed, and the spine coil; (3) with the track, patient bed, spine coil, and the base of the head coil; (4) with the track, patient bed, spine coil, and the whole head coil. PET data were acquired for 6 minutes under each configuration and were reconstructed by using the algorithm of time of flight (TOF) ordered subset expectation maximization (OSEM), with 20 subsets, 3 iterations, image matrix of 256 × 256, voxel size of 2.4 × 2.4 × 2.85 mm^3^, and a 3 mm Gaussian filter.

The attenuation maps for rigid objects were obtained from CT scans. It is also crucial for determining the relative position between these objects and the imaging FOV. The position of the track was fixed, so its attenuation map was hardcoded into reconstruction. The positions of patient bed and coils were determined by the axial position of patient bed since coils had a fixed position on patient bed. The position of the phantom was determined with an automatic coregistration process between the build-in model and the non-attenuation-corrected PET image.

**Figure 2:**
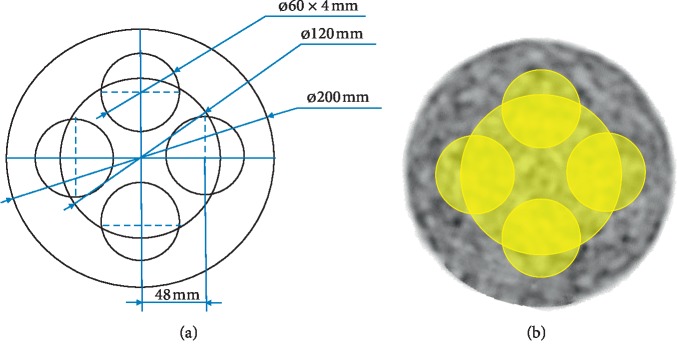
Schematic diagram showing region of interest drawing in cylinder phantom for uniformity calculation (a). Uniformity calculation on a PET image (b).

The uniformity of the image was calculated based on the following approach. Five slices located at the center, ±3 cm, and ±6 cm of the image were selected from the image volume. For each slice, mean SUVs from four circles with diameters of 60 mm and a circle with a diameter of 120 mm were measured, denoted as *B*1, *B*2, *B*3, *B*4, and *A*1, respectively (Figure 2). The slice uniformity is defined as

(1) $$\begin{array}{c} \text{slice}\_\text{uniformity}=\text{max}\left(\left|\frac{\max\left(B1,B2,B3,B4\right)-A1}{A1}\right|,\left|\frac{\min\left(B1,B2,B3,B4\right)-A1}{A1}\right|\right). \end{array}$$

The whole image uniformity is defined as the maximum of the slice uniformities from the five slices.

## 3. Patient Study

### 3.1. Ethical Statement

The patient study was retrospective and was approved by the Institutional Review Board (IRB)/Ethics Committee (IRB 88000039-QCN-CT5-01) in accordance with the ethical standards as laid down in the 1964 Declaration of Helsinki and its later amendments. Informed consent was obtained from all patients.

### 3.2. Patient Cohort

Thirty patients referred for 2-deoxy-2-[^18^F]fluoro-D-glucose (FDG) PET/CT scans were included. Inclusion criteria were as follows: (1) patients proven to have or suspected of having malignancy, with an indication for PET/CT for diagnosis, staging or restaging, follow-up, and therapy-response evaluation; (2) medical conditions of patients were stable. Exclusion criteria were as follows: (1) examinations were only on one bed position (e.g., head or abdomen); (2) obvious metal artifacts; (3) technical problems (e.g., patient movement); and (4) MR-CT registration failure. The basic information of patients is summarized in Table 1.

### 3.3. PET/CT Imaging

All patients fasted for at least 6 hours, and serum glucose was checked before the injection of FDG. The amount of injected radioactivity was calculated by measuring the radioactivity of the syringe before and after injection. The mean injected dose was 310.8 MBq (standard deviation (SD), 63.6). One hour after injection, PET/CT scanning was performed from the skull base to the proximal thigh in a supine position with arms over head, on the uMI 780 PET/CT scanner (United Imaging Healthcare, Shanghai, China) which had a similar PET configuration with the uPMR 790 PET/MRI scanner. Helical CT acquisition was performed without contrast enhancement using the following parameters: tube current, 274 mAs; tube voltage, 120 kV; collimation configuration, 80 × 0.5 mm; pitch, 0.516; matrix size, 512 × 512; and scanning time, 0.8 seconds per rotation. PET was acquired for 2 minutes per bed position in a three-dimensional mode, and images were reconstructed by using the OSEM algorithm. The image matrix was 256 × 256, corresponding to a 3 mm in-plane pixel size with a plane thickness of 3 mm.

### 3.4. PET/MR Imaging

After PET/CT imaging, all patients experienced simultaneous PET/MR imaging on the uPMR 790 PET/MRI system (United Imaging Healthcare, Shanghai, China), composed of a 3.0-Tesla MR imager and a fully integrated PET detector. Mean time intervals between PET/CT and PET/MR imaging was 45.4 (SD 16.2) minutes, while mean time intervals between FDG injection and PET/MR imaging was 101.7 (SD, 19.6) minutes (Table 1). For MRAC, a 3D T1-weighted spoiled gradient-echo sequence with Dixon-based water-fat separation imaging (WFI) was acquired in coronal plane with a repetition time of 4.6 ms, an echo time of 3.2 ms, a slice thickness of 2.4 mm, an FOV of 500 × 350 mm, a matrix of 206 × 144, and an acquisition time of 31 seconds. Compressed sensing-based technology was used to speed up the acquisition. Tissue segmentation and *μ*-map calculation were carried out automatically by the vendor-provided algorithm. Whole-body PET data were acquired in 3D list mode. Four bed positions with an average time of 6 minutes per bed were set to cover the area from skull base to proximal thigh of patients with an overlap of 30%.

## 4. PET Attenuation Correction

### 4.1. MR-Based Attenuation Correction

The WFI images were preprocessed to correct for the bias field signal before submitting to segmentation. Then, the corrected images were segmented into four classes: soft tissue, fat, lung, and air according to predetermined attenuation coefficients, namely, 0.096 cm^−1^, 0.080 cm^−1^, 0.032 cm^−1^, and 0 cm^−1^, in order. A deep learning-based technique was used for lung segmentation so that the segmentation could be done with arbitrary bed positions without users' input [18]. A U-Net model was used, and training data were made by arbitrarily cropping whole-body DIXON in-phase images with lung region labeled [19]. Furthermore, truncation completion was achieved with the contour of arms from PET images by segmenting non-attenuation-corrected PET images. PET images were reconstructed with a standard process provided by the vendor as follows: TOF-OSEM with 20 subsets, 3 iterations, image matrix of 256 × 256, voxel size of 2.4 × 2.4 × 2.85 mm^3^, and 3 mm post-Gaussian filter.

### 4.2. CT-Based Attenuation Correction

**Figure 3:**
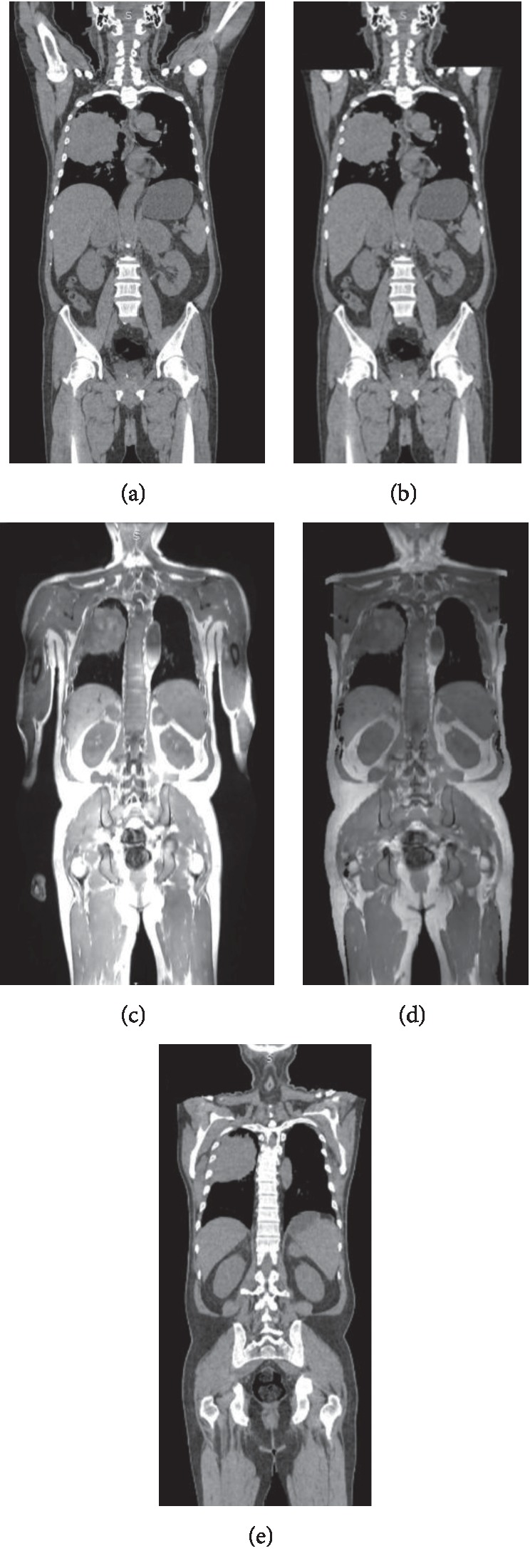
Coregistration process between MR and CT images. (a) Whole-body CT image; (b) CT image without arm; (c) whole-body MR image; (d) MR image without arm; (e) CT image that coregistered to MR image space.

In order to create CT *μ*-maps for PET reconstruction, the CT images were coregistered to the PET/MR space with a nonrigid registration algorithm using the publicly available software—Elastix [20]. The set of parameters were carefully adjusted so that the coregistration performance was optimized. We would like to point out that since nonrigid registration is underdetermined by nature, the algorithm primarily focused on good alignment in tissue boundaries between two modalities. Since CT images were acquired with arms up while MR images were acquired with arms down, the arms were excluded in the coregistration process with body-arm boundaries manually drawn for each case (Figure 3). After the coregistration process, the arms from MRAC were stitched back to CTAC to form a complete attenuation map. For each patient, two sets of CT *μ*-maps were obtained. One was derived directly from CT images with linear transformation. The other was segmented CT *μ*-maps, which was calculated as follows: for voxels of LAC <0.003 mm^−1^, they were assigned LAC = 0 mm^−1^ (air); for voxels of LAC 0.003–0.009 mm^−1^, they were assigned LAC = 0.008 mm^−1^ (fat); for voxels of LAC 0.009–0.011 mm^−1^, they were assigned LAC = 0.0096 mm^−1^ (water); for voxels of LAC > 0.011 mm^−1^, they were assigned LAC = 0.0161 mm^−1^ (bone); and for lung regions, they were assigned LAC = 0.0032 mm^−1^. The CTAC maps were used in the same manner as MRAC maps in the PET reconstruction process.

**Figure 4:**
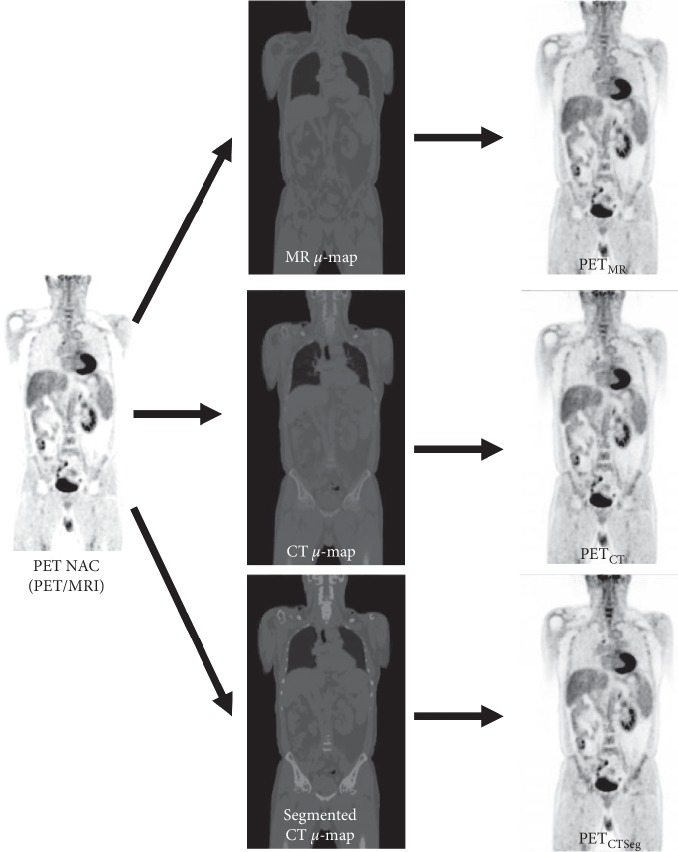
Workflow for the creation of the analyzed PET data from PET/MR corrected with MR-based (PET_MR_), CT-based (PET_CT_), and segmented CT-based AC (PET_CTSeg_).

In summary, the PET dataset from PET/MRI was reconstructed using the same parameters with the MR *μ*-map (PET_MR_), the CT *μ*-map (PET_CT_), and the segmented CT *μ*-map (PET_CTSeg_), as shown in Figure 4.

### 4.3. PET Quantification Analysis

Quantification analyses were conducted on the vendor-provided image viewer. For measuring SUVs on the 3 sets of PET data with the same regions of interest (ROIs), the Dixon T1 images were selected and registered with all of the PET data, respectively, for providing anatomic information. ROIs in diameters of 2 cm were drawn in homogeneous area of main organs—the right lobe of the liver, the right lower lobe of the lung, and the fourth lumbar vertebrae, and SUVs were measured. Care was taken to avoid placing ROIs on large vessels, organ borders, lesions, and close to the border of the PET FOV. In addition, for analyzing the accuracy of MRAC on lesion basis, 52 FDG-avid lesions from 30 patients were selected and grouped into two parts: (1) lesions located within or around bone (*n* = 28) and (2) lesions located in soft tissue (*n* = 24). VOIs with a 50% isocontour of SUV_max_ were drawn around lesions. Mean SUVs (SUV_mean_) were measured for major organs; while both SUV_mean_ and maximum SUV (SUV_max_) were measured for lesions. This work was performed by a nuclear medicine physician with experience over 15 years.

### 4.4. Statistical Analysis

Statistical analyses were performed using SPSS 20.0 (SPSS Statistics; IBM, Armonk, NY). Pearson correlation coefficient was calculated to assess the consistency between MRAC and CTAC. For each ROI or VOI, the relative difference of SUV in percent between PET_CT_ and PET_MR_ was defined as (SUV_MR_–SUV_CT_)/(SUV_CT_), and the relative difference of SUV between PET_CTseg_ and PET_MR_ was defined as (SUV_MR_–SUV_CTSeg_)/(SUV_CTSeg_). Intergroup SUV differences were analyzed using one-way ANOVA with Bonferroni post hoc correction. The significant level was set as *P* value less than 0.05 for all statistical analyses.

## 5. Results

### 5.1. MRAC Accuracy under Different MR Hardware Configurations

Images of cylinder phantom and calculated uniformities under different MR configurations are illustrated in Figure 1. Good image uniformities (<9%) could be obtained in all images with different MR hardware configurations. Uniformities under all MR hardware configurations met the system requirement, which was defined as <10%, demonstrating accurate MRAC for MR hardware in uPMR 790 system.

### 5.2. Comparisons of SUVs in Main Organs

Mean SUVs of bone, liver, and lung all revealed high correlations (>0.95) either between PET_CT_ and PET_MR_, or between PET_CTSeg_ and PET_MR_, demonstrating good consistencies of SUV quantifications between MRAC and CTAC (Table 2).

The mean relative differences of SUV_mean_ in normal bone, liver, and lung were −18.8% (SD, 5.1%), −8.0% (SD, 3.8%), and −12.2% (SD, 4.6%), respectively, between PET_CT_ and PET_MR_, and −19.0% (SD, 8.3%), −3.5% (SD, 3.6%), and −3.3% (SD, 6.0%), respectively, between PET_CTSeg_ and PET_MR_. These differences were significant for all organs (*P* < 0.05). The greatest underestimation in PET_MR_ was found in bone with a relative difference of −18.8 ± 5.1% between PET_MR_ and PET_CT_, and −19.0 ± 8.3% between PET_MR_ and PET_CTseg_. The absolute intergroup differences between PET_MR_ and PET_CT_ were significantly larger than those between PET_MR_ and PET_CTSeg_, both in liver and in lung (*P* < 0.001), but not in bone (*P*=0.893, Table 2).

### 5.3. Comparisons of SUVs in Lesions

Of the 28 bone lesions, both mean and maximum SUVs showed high correlations (*r* > 0.93) between PET_MR_ and PET_CT_ and between PET_MR_ and PET_CTSeg_, demonstrating good consistencies between MRAC and CTAC (Table 2). The relative differences of SUV_mean_ were −18.1 ± 17.4% between PET_MR_ and PET_CT_, and −19.3 ± 20.4% between PET_MR_ and PET_CTSeg_, while the relative differences of SUV_max_ were −13.3 ± 10.5% between PET_MR_ and PET_CT_, and −17.9 ± 15.1% between PET_MR_ and PET_CTSeg_; all of these differences were statistically significant (*P* < 0.001, Table 2). Segmentation to CTAC had no effect on SUV quantification, as intergroup differences between PET_MR_ and PET_CT_ were not significantly different from those between PET_MR_ and PET_CTSeg,_ either for SUV_mean_ or for SUV_max_ (*P*=0.519 and 0.217, respectively; Table 2).

An even higher correlation was found in the group of twenty-four lesions in soft tissue. The correlation of SUV_mean_ and SUV_max_ between PET_MR_ and PET_CT_ (*r* > 0.98) and between PET_MR_ and PET_CTSeg_ (*r* > 0.97) demonstrated the excellent consistencies between MRAC and CTAC (Table 2). The relative differences of SUV_mean_ were measured −8.2 ± 5.8% between PET_MR_ and PET_CT_, and −5.5 ± 6.3% between PET_MR_ and PET_CTSeg_, whereas the relative differences of SUV_max_ were −7.3 ± 3.6% between PET_MR_ and PET_CT_, and −4.4 ± 4.0% between PET_MR_ and PET_CTSeg_. All these differences were statistically significant (*P* < 0.001). In addition, the absolute intergroup differences between PET_MR_ and PET_CT_ were significantly larger than those between PET_MR_ and PET_CTSeg_, both in SUV_max_ and in SUV_mean_ (*P* < 0.001, Table 2), indicating a significant impact of segmentation to CT *μ*-map on quantification differences between MRAC-based PET and CTAC-based PET.

## 6. Discussion

In this study, we validated the quantitative performance of a newly released simultaneous PET/MRI (uPMR 790, United Imaging Healthcare, Shanghai, China). The system presented good image uniformity in phantom measurements for a variety of MR hardware configurations, which demonstrated the accuracy of MRAC for a variety of MR hardware options. A systematic clinical comparison showed that SUVs from PET_MR_, PET_CT_ and PET_CTSeg_ were highly correlated with each other, either from a normal tissue basis or from a lesion basis. In addition, the relative differences of SUV between MRAC-based PET and CTAC-based PET were generally less than <10%. These results indicated that the uPMR 790 system functioned with great promise to provide PET images with accurate quantification that was consistent with PET/CT. This system opens many opportunities for clinical and research utility for the large amount of Chinese population.

There are many technical challenges in the physical integration of simultaneous PET/MR systems. One significant challenge is the hardware components, including the patient bed, the track, and the MR coils. The strong radiofrequency (RF) wave and the rapid switching gradient system may induce noise to the PET data. On the other hand, the highly sensitive MR RF may pick up noise from the digital circuit of PET. If the coil is not accounted for in the attenuation map, significant quantification bias may occur, which can be as high as 20% [21–23]. However, in this study, good image uniformities and quantitative accuracies (bias < 10%) were obtained in all images with different MR hardware configurations, part of which had not been discussed previously. This result was close to the data (5.2%–8.2%) reported by Wollenweber et al. [22], who advocated that the bias introduced by the coil was less susceptible to cause clinically relevant errors. We did not study the bias caused by flexible surface coil, as it had been reported to be minimal [22, 24].

The segmented MRAC method classifies the body into different types of tissue; however, the classification of bone is typically underestimated and therefore not implemented into the *μ*-map, whereas in CTAC, bone causes the highest attenuation and is easily accounted for. Therefore, significant underestimation of PET_MR_ in bone is expected. Eiber et al. [11], Hershah et al. [12], and Heusch et al. [13] found an underestimation of SUVs (−30.1% on average) in PET/MR for bones. However, as these studies were performed between PET/CT and a subsequent PET/MRI, the results might be biased from the impact of the biological clearance of the tracer. When discussing the impact of different AC methods on SUV in bone or bone lesions in the same PET dataset, Martinez-Möller et al. [7] and Eiber et al. [25] found relatively lower influences, with an average underestimation of up to −13%. Seith et al. [9] observed a maximum SUV underestimation of −17.3% in PET/MR in bone lesions. Our results regarding the underestimation of SUVs in normal bone (−18.8%) and bone lesions (−18.1% for SUV_mean_ and −13.3% for SUV_max_) were close to these reported data. One may argue that this underestimation can lead to misinterpretation in examinations performed for assessing bone lesions. However, it should be noted that the normal uptake of FDG in normal bone is typically low, and therefore, even a small underestimation of PET_MR_ can cause a large deviation in percentage.

For soft tissue and soft-tissue lesions, the underestimations of PET_MR_ were relative low (−8.0% for normal liver; −8.2% of SUV_mean_ and −7.3% of SUV_max_ for soft-tissue lesions), as compared to bone tissue. Similar results were reported in previous studies which demonstrated underestimations in SUV of up to −11% [9, 26, 27].

Another area with substantial underestimation of PET_MR_ was lung (−12.2 ± 4.6%). Previous studies indicated that the segmentation-based MRAC might not be sufficient for reliable SUV quantification in the lung, as MRAC ignored the heterogeneity of lung intensity, probably caused by gravitation [9, 10, 13, 28, 29]. Both overestimation [9, 28] and underestimation [10, 28] of SUV quantification in PET_MR_ in or adjacent to lung area had been reported. Seith et al. demonstrated an overestimation of SUV (5.7 ± 13.0%) in the anterior parts and an underestimation (−14.0 ± 15.8%) in the posterior parts of the lungs [28]. This partially supported the result of the current study, as the right lower lobe of the lung was selected for SUV measurement in this study.

One interesting finding in this study was that the relative differences of SUV compared directly between segmented MRAC-based PET (PET_MR_) and linear-CTAC-based PET (PET_CT_) were larger than those between PET_MR_ and PET_CTSeg_ when both attenuation corrections were segmentation based. This phenomenon was substantial in soft tissue or soft-tissue lesions (*P* < 0.001; Table 2). This means the segmentation nature of MRAC might have partially contributed to the deviations of SUV quantifications between PET_MR_ and PET_CT_. But the changes from differences of SUV between PET_MR_ and PET_CT_ to differences between PET_MR_ and PET_CTSeg_ were not significant, either in bone or in bone lesions. The underlying reason might be associated with the inability of MRAC in the identification of bone. In recent years, new techniques have been developed for MRAC with continuous LACs for bone leading to improved quantification in bone and bone lesions [30, 31]. These methods could reduce bone-related PET biases and should be used in future for whole-body MRAC.

There are several limitations in this study. First, areas of patient arms were excluded from the analysis. Since PET/CT were acquired with arms up while PET/MRI were acquired with arms down, it is very difficult to compare MRAC and CTAC in these areas. We note that none of the lesions analyzed and none of the main organs selected were in these areas. Second, ACs of brain PETs were not evaluated because the special anatomical characteristics and the different diagnostic demands in the brain were beyond the scope of this study.

## 7. Conclusion

The simultaneous whole-body PET/MRI uPMR 790 system released by the United Imaging Healthcare Corporation (Shanghai, China) functioned with accurate attenuation corrections and SUV quantifications. The SUV deviations were generally less than 10%, compared to PET/CT. This system opens many opportunities for clinical and research utility for the large amount of Chinese population. However, in bone, SUV underestimations can be substantial, which may be partially due to the segmentation of the MR-based *μ*-maps.

## Figures and Tables

**Table 1:** Basic information of patients.

Patient	Sex	Age (years)	Height (cm)	Weight (kg)	Diagnosis	Dose (mCi)	Duration of PET/MRI after injection (min)	Clinical indication
1	F	55	162.0	62.0	Lung cancer	7.39	61	Follow-up
2	F	71	152.0	70.0	Cerebral metastasis	8.33	98	Diagnosis
3	M	57	172.0	68.0	Rectal cancer	8.65	114	Restaging
4	M	65	180.0	87.5	Lymphoma	10.49	99	Staging
5	M	64	170.0	64.0	Esophageal cancer, lung cancer	8.22	104	Staging
6	M	66	167.0	76.0	Pancreatic cancer	9.52	146	Staging
7	M	17	174.0	72.0	Thyroid cancer	8.60	94	Diagnosis
8	M	69	162.0	67.0	Enterocoelic sarcoma	8.65	120	Staging
9	F	73	157.0	63.0	Ovarian cancer	7.05	96	Staging
10	F	50	165.0	54.0	Pancreatic cancer	6.99	87	Staging
11	F	65	163.0	58.0	Cholangiocarcinoma	6.87	101	Staging
12	M	66	164.0	51.0	Esophageal cancer	6.13	102	Staging
13	F	51	161.0	67.0	Rectal cancer	8.01	90	TRE
14	F	68	161.0	63.0	Lung cancer	7.33	87	Staging
15	M	46	170.0	105.0	Liver cancer	13.61	162	Diagnosis
16	M	62	175.0	75.0	Lung cancer	9.33	88	Staging
17	M	70	170.0	70.0	Duodenal cancer	8.82	96	Staging
18	M	70	162.0	69.0	Lung cancer	8.20	119	Staging
19	M	64	164.0	61.0	Colon cancer	8.50	117	Staging
20	M	63	168.0	68.0	Gastric cancer	8.57	94	TRE
21	M	65	172.0	71.0	Colon cancer	8.85	107	Staging
22	M	69	170.0	72.0	Pancreatic cancer	9.99	115	Diagnosis
23	M	54	151.0	52.0	Liver cancer	6.40	66	Staging
24	M	46	173.0	84.0	Liposarcoma in teres major	10.71	103	Staging
25	M	60	171.0	73.8	Liver cancer	3.64	94	TRE
26	M	75	173.0	74.0	Liver cancer	9.38	107	Restaging
27	F	60	156.0	56.0	Duodenal cancer	6.69	114	Staging
28	M	51	174.0	85.0	Liver cancer	10.08	93	Diagnosis
29	M	61	172.0	70.5	Lymphoma	8.45	82	Diagnosis
30	F	69	156.0	60.0	Liver cancer	8.63	95	Diagnosis
Mean		60.7	166.2	69.0		8.40	101.7	
SD		11.2	7.1	11.1		1.72	19.6	

Note: M, male; F, female; SD, standard deviation; TRE, therapy-response evaluation.

**Table 2:** Correlations of SUVs from different PET images in major organs and in selected lesions.

SUVs	PET_MR_ and PET_CT_			PET_MR_ and PET_CTSeg_			*P*3
	Rho	RD1	*P*1	Rho	RD2	*P*2	
SUV_mean_ of main organs							
Bone	0.985	−18.8 ± 5.1%	<0.001	0.967	−19.0 ± 8.3%	<0.001	0.893
Liver	0.973	−8.0 ± 3.8%	<0.001	0.949	−3.5 ± 3.6%	<0.001	<0.001
Lung	0.983	−12.2 ± 4.6%	<0.001	0.959	−3.3 ± 6.0%	0.010	<0.001
Lesions within or around bone							
SUV_mean_	0.947	−18.1 ± 17.4%	<0.001	0.938	−19.3 ± 20.4%	<0.001	0.519
SUV_max_	0.997	−13.3 ± 10.5%	<0.001	0.975	−17.9 ± 15.1%	<0.001	0.217
Lesions in soft tissue							
SUV_mean_	0.981	−8.2 ± 5.8%	<0.001	0.979	−5.5 ± 6.3%	<0.001	<0.001
SUV_max_	0.996	−7.3 ± 3.6%	<0.001	0.996	−4.4 ± 4.0%	<0.001	<0.001

Note: SUV, standardized uptake value; max, maximum; PET_MR_, MR attenuation correction-based positron emission tomography; PET_CT_, PET images from PET/MRI that reconstructed from CT *μ*-map; PET_CTSeg_, PET images from PET/MRI that reconstructed from segmented CT *μ*-map; Rho, Pearson's correlation coefficient; RD, relative differences; *P*1 and *P*2 were statistical *P* values of RD1 and RD2, respectively; *P*3, statistical *P* values of paired *t*-test between RD1 and RD2.

## Data Availability

Relevant data can be accessed through proper request, from the first author.
